# Management of Multiple Acyl-CoA Dehydrogenase Deficiency (MADD) in Pregnancy

**DOI:** 10.3390/metabo15070432

**Published:** 2025-06-24

**Authors:** Matthew A. Shear, Allie LaTray, Irene J. Chang, Annalisa Post, Renata C. Gallagher

**Affiliations:** 1 Division of Maternal-Fetal Medicine and Reproductive Genetics, Department of Obstetrics, Gynecology, & Reproductive Sciences, University of California, 490 Illinois Street, Box 0132, San Francisco, CA 94143, USA; 2 Division of Medical Genetics, Department of Pediatrics, University of California, San Francisco, CA 94143, USA

**Keywords:** inborn error of metabolism, maternal-fetal medicine, pregnancy, biochemical genetics

## Abstract

Multiple acyl-CoA dehydrogenase deficiency (MADD), also known as glutaric acidemia/glutaric aciduria type II (GA II), is an inborn error of fatty acid, amino acid, and choline metabolism. The chronic management of MADD involves both dietary fat and protein restriction to reduce the substrates of the dehydrogenases affected, the avoidance of prolonged fasting as in any fat metabolism disorder, and monitoring for potential complications. Due to its rarity, there is little published experience on the management of MADD in pregnancy. Herein, we report the successful management of a pregnancy in a patient with late-onset or type III MADD, with considerations for preconception, antepartum, intrapartum, and postpartum care.

## 1. Introduction

Multiple acyl-CoA dehydrogenase deficiency (MADD), also known as glutaric acidemia/glutaric aciduria type II (GA II), is an inborn error of fatty acid, amino acid, and choline metabolism. It can present from infancy to adulthood with life-threatening metabolic disarray, including metabolic acidosis, hyperammonemia, and hypoglycemia. MADD is an autosomal recessive disorder caused by homozygous or compound heterozygous DNA variants in either the *ETFA*, *ETFB*, or *ETFDH* genes. These genes encode the protein subunits of the mitochondrial electron transfer flavoprotein and the electron transfer flavoprotein (ETF) dehydrogenase, which facilitate electron transfer from the dehydrogenases involved in the metabolism of both fats and proteins to the mitochondrial electron transport (respiratory) chain. The majority of affected individuals harbor pathogenic variants in *ETFDH*, which results in a deficiency in the mitochondrial enzyme ETF-ubiquinone oxidoreductase and the abnormal relay of electrons to complex III within the respiratory chain [1,2,3].

The characteristic biochemical testing pattern for MADD includes elevations in multiple short-, medium-, and long-chain acylcarnitine species and elevations in specific urine organic acids and urine acylglycines that are characteristic of the multiple dehydrogenases that interact with ETF and ETF-DH. These include glutaric acid, characteristic of glutaryl-CoA dehydrogenase deficiency, leading to the alternate name for this disorder, glutaric aciduria type II. Even in mild cases, biochemical abnormalities can be seen on urine and blood testing [4], and, in very mild cases, they may also be missed on metabolic testing. Diagnosis is typically confirmed molecularly with biallelic variants identified in one of the three causative genes (*ETFA*, *ETFB*, or *ETFDH*), and enzyme testing may also be performed [3].

Clinically, MADD presents with a broad phenotypic spectrum of severity and is subdivided into a neonatal-onset form with or without congenital anomalies (type I or type II, respectively) and a late-onset form (type III) [5]. Patients with type I or II may have dysmorphic facial features such as hypoplastic midface and a high forehead, abdominal wall defects, and renal cysts with enlarged kidneys [6]. Hypospadias, chordee, and central nervous system heterotopias have also been associated [3]. Clinical presentation in the neonatal period may include metabolic crisis with hypoglycemia, hyperammonemia, and acidosis. If initial treatment is successful and a diagnosis is made, survivors are at risk of recurrent metabolic decompensation, particularly in illness, and are at risk of complications, including hypertrophic with progression to dilated cardiomyopathy. Patients with type III typically present with exercise intolerance due to skeletal muscle myopathy and myalgias; rhabdomyolysis with metabolic crisis associated with exertion is also possible, albeit less common [3].

The chronic management of MADD involves both dietary fat and protein restriction to reduce the substrates of the dehydrogenases affected, the avoidance of prolonged fasting as in any fat metabolism disorder, and monitoring for potential complications. Riboflavin supplementation has been shown to be efficacious, particularly in type III MADD. Riboflavin is converted to flavin adenine dinucleotide (FAD), which is a cofactor for both ETF and ETFDH, and stabilizes the ETFDH enzyme and enhances its activity [7,8]. Free carnitine may be low due to the conjugation of carnitine with accumulating metabolites, and carnitine supplementation may be provided. Due to the risk of metabolic decompensation with illness, patients should have both a sick day plan that includes increased carbohydrates and an emergency letter directing emergency room care in illness. Fasting for surgery and other procedures and the stress of surgery can provoke metabolic decompensation, and patients should also have a tailored plan for fasted procedures including surgery that provides calories in the form of oral glucose before and IV glucose during the procedure. Pregnancy, labor, delivery, and the postpartum period may pose risks to patients with MADD.

Due to its rarity, there is little published experience on the management of MADD in pregnancy [9,10]. Case reports from 2012 and 2017 document successful pregnancies leading to live births; however, both patients underwent a planned elective cesarean to avoid the risk of metabolic decompensation during labor and delivery [10]. Herein, we report the successful management of a pregnancy in a patient with late-onset or type III MADD, with considerations for preconception, antepartum, intrapartum, and postpartum care.

## 2. Case Report

Our patient initially presented at age 21 years with a seizure and coma. This was erroneously attributed to substance use and no alternative etiology was pursued. She recovered with supportive care and was discharged after 9 days of admission without a metabolic diagnosis. At age 26, she was admitted for an episode of rhabdomyolysis with peak creatine kinase (CK) 4462 U/L. A muscle biopsy demonstrated pauci-inflammatory necrotizing myopathy with lipid accumulation and the absence of ragged red fibers. magnetic resonance imaging (MRI) of the brain and spine was unrevealing. Over the following six-month period, the patient had recurrent episodes of pain and weakness, elevated CK, hypothermia and hypoglycemia that were unprovoked by exercise or illness. This progressed to a metabolic crisis and the patient was next “found down”, admitted to the ICU with severe hypoglycemia, non-anion-gap metabolic acidosis, episodic hypothermia, rhabdomyolysis (peak CK 36,155 U/L), transaminitis, cardiomyopathy with moderate global biventricular systolic dysfunction with normal biventricular size and ejection fraction (EF) 30%, and renal failure with anuria. Laboratory testing was notable for a pH nadir of 7.0, bicarbonate of 25 mmol/L, lactate peak at 8.1 mmol/L, and blood glucose nadir of 16 mg/dL.

Neurology, internal medicine, critical care medicine, and infectious disease were consulted for the treatment of multi-organ failure and presumed sepsis. Biochemical testing included an acylcarnitine profile that demonstrated low carnitine free and total levels and elevations in long-chain acylcarnitine species including C16 and C18:1, suggestive of CPT II deficiency or carnitine-acylcarnitine translocase (CACT) deficiency. Treatment was initiated with carnitine, the avoidance of prolonged fasting, IV therapy with dextrose, and a low-fat and high-carbohydrate diet and MCT oil, and she recovered and was discharged. *CPT2* and *SLC25A20* gene sequencing with deletion/duplication analysis was negative (Baylor). A fatty acid oxidation probe assay was negative (Mayo), although riboflavin had been added to the media, which may have affected the results. Exome trio sequencing (University of California, Los Angeles Molecular Diagnostic Laboratory) identified compound heterozygous variants in *ETFDH* (NM_004453.2: c.1367 C>T p.Pro456Leu pathogenic and NM_004453.2 c.1487T>C p.Leu496Pro likely pathogenic), resulting in a change in her diagnosis to MADD. Subsequent acylcarnitine profiles and urine organic acids were consistent with MADD and not CPTII or CACT deficiency, and MCT oil was discontinued. The patient continued in a stable condition and was managed outpatient with riboflavin, carnitine supplementation, and reduced dietary fat and protein intake (Table 1). She had regular metabolic clinic visits and monitoring laboratories, had a sick day plan and an emergency letter, and had regular cardiac monitoring. Her cardiac status returned to normal at 1 month after hospital d/c. She maintained a job, participated in regular exercise, and married.

## 3. Preconception

The patient was referred to maternal–fetal medicine at 33 years of age for preconception counseling. The risks of pregnancy were reviewed, including the precipitation of metabolic decompensation, the development of hypertensive disorders of pregnancy, and the need for careful dietary management. The patient’s partner was recommended for prenatal genetic testing with sequencing of *ETFDH* to evaluate the procreative genetic risk. Both were offered expanded genetic carrier screening, which returned negative results for paternal variants in *ETFA*, *ETFB*, or *ETFDH* [11]. Prenatal diagnosis with fetal sequencing for *ETFDH* via amniocentesis or chorionic villus sampling was offered given the risk of de novo variants. The patient was initiated on a prenatal vitamin containing folate and DHA, instructed to continue riboflavin and carnitine, and subsequently conceived spontaneously. Antepartum care was undertaken with a multidisciplinary team including cardiology, obstetric anesthesia, maternal–fetal medicine, metabolic genetics, and metabolic dietitians. The patient was again advised to avoid prolonged fasting and her diet was monitored carefully. A baseline electrocardiogram (ECG) was obtained and multimodal antiemetics were prescribed prophylactically for use in the first trimester as needed. A glucose polymer formula was refilled to be available for use during illness. The Institute of Medicine guidelines were reviewed, recommending a total weight gain during pregnancy of 11.5–16 kg based on the patient’s pre-pregnancy body mass index (BMI) of 20.4 kg/m^2^ [12].

## 4. Antepartum Care

The prescribed diet at the beginning of the second trimester included 1900–2100 kcal per day with fat restriction to 30% of the total calories. The protein allowance was increased to 1.1–1.2 g of protein per kilogram to accommodate fetal growth and adjusted according to the laboratory results, with the balance of calories derived from fat, protein, and carbohydrates as noted in Table 1. Additional calcium and vitamin D were started to meet the daily recommended intake (DRI). Biochemical and nutritional laboratory parameters were monitored, including urine organic acids, plasma total and free carnitine, zinc, vitamin D, vitamin B12, selenium, quantitative amino acids, prealbumin, iron, the acylcarnitine profile, the fatty acid profile, and urine acylglycines. Compliance with carnitine and riboflavin was encouraged. Acylcarnitine species and urine organic acid analytes were stable, and diet adherence was good. Additional zinc and iron supplementation was required based on low values. All diet records were reported during clinic visits and were analyzed with nutritional analysis from Food Processor® Software (11.11.0), ©(2022) ESHA Research, Inc. (Salem, OR, USA).

To increase the protein intake in the second trimester to support fetal growth, additional supplements including protein bars and shakes were initiated to meet the higher protein demands of pregnancy and prevent branched-chain amino acid deficiencies. Slightly higher calories were prescribed in the third trimester with the goal of 2000–2200 kcals per day. The percent fat restriction was recommended to remain the same, but an analysis of the patient’s diet demonstrated reduced compliance with fat restriction, with a higher proportion of calories from fat. The essential fatty acid profiles were analyzed and returned reassuring results, without evidence of deficiencies. Protein restriction was maintained at 1.1–1.2 g protein per kilogram to maintain normal prealbumin, branched-chain amino acids, and other laboratory parameters. By the end of the third trimester, the patient reported difficulty in meeting her caloric and protein goals and relied on protein supplements and higher-protein and higher calorie foods (e.g., meats, eggs) to meet the daily goals due to reports of early satiety. A summary of the patient’s dietary management is included in Table 1.

## 5. Labor and Delivery Metabolic Management

The patient’s blood pressure, antenatal testing, fetal growth ultrasounds, and serum laboratory testing including liver function tests remained reassuring and within the normal limits. Due to the need for careful metabolic management, including metabolic nutritional management in labor and delivery, scheduled induction was recommended. The patient presented for a scheduled induction of labor at 40w0d gestation following shared decision-making [13,14]. A detailed metabolic management plan for labor and delivery and postpartum was created and posted to the patient’s chart and sent to the obstetric team in advance (Table 2). Due to the patient’s history of cardiomyopathy, a baseline laboratory analysis was obtained at admission, including a complete metabolic profile (CMP) and creatinine phosphokinase (CPK), which were normal. A 12-lead ECG was obtained and the patient was placed on continuous cardiac telemetry. The overall management goals during labor and delivery included dietary maintenance and the provision of sufficient calories and protein to minimize protein and fat catabolism, close laboratory and cardiac monitoring, and the provision of adequate calories and protein as IV dextrose and IV amino acids while NPO (Table 2).

The induction of labor was undertaken using routine obstetric interventions. At 2 cm of cervical dilation, the patient underwent the uncomplicated placement of neuraxial anesthesia. While NPO following epidural placement, she was started on IV dextrose, IV carnitine, and protein supplementation with IV Clinimix (4.25% amino acids/5% dextrose)™ (Table 2). One hour following epidural placement, an acute episode of fetal bradycardia to 60 beats per minute was noted. Routine obstetric maneuvers were employed, including sublingual nitroglycerine administration for uterine relaxation, fluid bolus, and maternal repositioning. Without an improvement in the fetal bradycardia, the patient was taken to the operating room, where an urgent cesarean delivery was performed. During surgery, the patient was found to have hypertensive urgency and diagnosed with preeclampsia with severe features and started on magnesium IV for seizure prophylaxis using routine obstetric dosing. A liveborn female infant emerged vigorous with Apgars of 8 and 9 at 1 and 5 min, respectively, and a birthweight of 3360 g. There were no neonatal complications, and her newborn screening was normal.

## 6. Postpartum

Following delivery, the patient developed hypothermia to 32 °C, underwent evaluation for sepsis which was negative, and was managed with warming blankets. The patient’s postpartum and postoperative course was otherwise unremarkable. The patient was placed on a pathway for enhanced recovery after surgery (ERAS) [15]. IV Clinimix (4.25/5%), IV dextrose, and IV carnitine were continued at their prescribed doses and the patient’s diet was advanced immediately. By postoperative day 1, the patient was tolerating a full regular diet and IV Clinimix and dextrose were discontinued, along with a transition to oral carnitine. Her blood pressure remained controlled with a single daily dose of nifedipine. The metabolic lab assessment, including CK and CMP, remained reassuring. She underwent a lactation consultation and was breastfeeding successfully. She was discharged on postoperative day 4.

## 7. Discussion

Multiple acyl-CoA dehydrogenase deficiency (MADD) is a rare genetic disorder of fatty acid, amino acid, and choline metabolism with an estimated incidence at birth of 1:250,000 [3], with little published experience regarding successful pregnancy management [9,10].

Patients with MADD are at risk for metabolic decompensation during periods of catabolism, including pregnancy, labor, delivery, and the postpartum period. Cardiomyopathy and skeletal myopathies are also known complications seen in MADD. To compensate for the increased cardiac demand, blood volume, and metabolic demand of pregnancy, patients with MADD will likely benefit from enhanced surveillance and cardiac monitoring. Despite the known risks and given that pregnancy and labor represent periods of increased metabolic challenge, there is no clear consensus regarding management.

Maintaining adequate caloric intake can be compromised by a number of factors unique to pregnancy. Nausea and vomiting in pregnancy affect 50–80% of pregnancies, typically in the first trimester. At its most severe, this may develop into hyperemesis gravidarum in about 3%, associated with weight loss and catabolism, which may trigger metabolic decompensations in MADD [16]. The use of multimodal antiemetics may help patients to maintain adequate oral intake, and the availability of IV hydration and metabolic consultation during early pregnancy is essential. Given the risk of cardiac arrhythmias and QT interval prolongation associated with some antiemetics, a baseline electrocardiogram (ECG) may be considered due to the cardiac risks associated with MADD. In the third trimester, early satiety due to the physical displacement of the intraabdominal anatomy secondary to the gravid uterus may also require dietary adjustment to frequent smaller meals and the liberal use of snacks and protein supplements to ensure adequate caloric intake and prevent protein deficiencies in MADD.

The dietary management of MADD in pregnancy balances increases in protein, lipids, and micronutrients needed to support the developing fetus [17] against the overall goals of protein and fat restriction and avoiding catabolism. Typical increases of 340 kcal in the second trimester and 450 kcal in the third trimester are needed, but patients may have varying needs based on their pre-pregnancy weight, weight trajectory, and target weight gain as per the Institute of Medicine guidelines [12]. Pregnant patients and their metabolic dietitians must also navigate additional obstetric dietary and lifestyle guidelines that include the avoidance of deli meat and unpasteurized dairy products to prevent listeriosis, the avoidance of undercooked meat or unwashed raw vegetables to prevent toxoplasmosis, the avoidance of fish with the highest mercury concentrations [18], and reducing exposure to toxic environmental agents by avoiding nonstick cookware and certain food storage containers [19]. Regular assessment of patients’ weight, lab monitoring of serum essential amino acids and micronutrients, and consultation with a metabolic dietitian can aid teams in maintaining appropriate caloric intake.

Individuals with MADD are at risk for free carnitine deficiency, which is also a physiologic feature during pregnancy, particularly in the third trimester [20]. Increased supplementation with L-carnitine helps maintain free plasma carnitine at normal or near normal levels, requiring an. increased dosage in the third trimester, when the free carnitine levels are lower.

Labor and delivery represent the period of the highest metabolic demand in pregnancy. Studies of hemodynamic changes in pregnancy demonstrate a sharp rise in cardiac output in the first trimester, with approximately a 45% increase in the plasma volume by term [21]. The metabolic demand continues to increase into the third trimester and peaks during the second stage of labor. Owing to uterine contractions, circulating catecholamines released from pain, and vigorous expulsive efforts, the basal metabolic demand may be increased in excess of 80% above pre-pregnancy levels. To compound this, emesis is common in active labor, and most centers in the US require the diet be limited to clear liquids following the placement of epidural analgesia. Ultimately, this may lead to a period that is vulnerable to catabolism, as the peak metabolic demand coincides with the nadir of caloric intake. Approaches to bridge this window among patients with FAODs include the liberal bedside availability of glucose-containing fluids, the continuation of L-carnitine either PO or IV, and the administration of protein- and dextrose-containing IV fluids for caloric supplementation. Delivery at a facility with the immediate availability of these interventions is recommended.

Postpartum also presents unique metabolic challenges for patients with FAODs. Lactation is known to increase the metabolic demand by up to 25%, requiring an additional 400–500 kcal/day [22]. Cesarean delivery is associated with a longer period of NPO, along with higher rates of obstetric morbidity that may contribute to metabolic stress [23,24]. Following surgery, increased protein intake is recommended for wound healing. Functional GI dysmotility or ileus may develop and can be compounded by narcotic use. Caring for a newborn disrupts parental sleep and meal schedules. The sleep and eating schedules of new parents may not coincide with standard hospital cafeteria hours, and coordination with the hospital food service to provide a 24/7 meal service may be required. Postpartum depression, affecting one out of seven women, also contributes to alterations in appetite and eating habits, necessitating careful screening for peripartum mood disorders [24,25].

## 8. Conclusions

Pregnancy among patients with MADD requires multidisciplinary management including metabolic genetics, maternal–fetal medicine, obstetric anesthesia, and metabolic dietitians. The principles of management include careful laboratory monitoring of the nutritional status, tailored dietary management, the treatment of conditions that may precipitate catabolism, careful monitoring for the development of preeclampsia, and the use of IV dextrose and IV amino acids or TPN during labor and delivery. Experience at our center among patients with a variety of fatty acid oxidation disorders supports attempts at a vaginal birth, while reserving cesarean delivery for usual obstetric indications. Communication between metabolic genetics and obstetric providers is critical, and consideration should be given to the development and documentation of customized and personalized management strategies in advance of delivery. A multidisciplinary team with appropriate expertise in metabolic genetics and high-risk obstetrics is necessary for the management of patients with complex inborn errors of metabolism such as MADD, who are at high risk for decompensation due a variety of metabolic and obstetric factors.

## Figures and Tables

**Table 1:** Nutritional management for a pregnancy complicated by maternal MADD.

Weight (kg)	Gestational Age (Weeks)	Calories (Total)	Protein (g/d) % Total Kcals	Fat (g/d) % Total Kcals	CHO (g/d) % Total Kcals	Micronutrients and Supplements
61	Preconception	Recommended: 1600–1800 Actual: 1630	Recommended: 0.8–1.0 g/kg/d Actual: 60 15%	Recommended: 30% Actual: 87 47%	Recommended: n/a Actual: 155 38%	Calcium 500 mg BID, carnitine, riboflavin, B12, prenatal vitamin (PNV) w/400 mcg folate and DHA
63	16	Recommended: 1900–2100 Actual: 1740	Recommended: 1.1–1.2 g/kg/d Actual: 46 10.5%	Recommended: 30% Actual: 86 44.5%	Recommended: n/a Actual: 195 45%	PNV, 600 mg calcium, Vit D 1000 IU, choline, carnitine 500 mg, riboflavin 400 mg BID
64.4	25	Recommended: 1900–2100 Actual: 1980	Recommended: 1.1–1.2 g/kg/d Actual: 86 17%	Recommended: 30% Actual: 90 41%	Recommended: n/a Actual: 207 42%	PNV, calcium 600 mg, Vit D 1000 IU, choline, carnitine 500 mg, riboflavin 400 mg BID, zinc 7.5 mg
71.2	30	Recommended: 2000–2200 Actual: 2000	Recommended: 1.1–1.2 g/kg/d Actual: 95 19%	Recommended: 30% Actual: 105 47%	Recommended: n/a Actual: 169 34%	PNV, 600 mg calcium, Vit D 1000 IU, choline, carnitine 500 mg, riboflavin 400 mg BID, Fe 15 mg (every other day)
74.8	34	Recommended: 2000–2200 Actual: 1600	Recommended: 1.1–1.2 g/kg/d Actual: 86 21.5%	Recommended: 30% Actual: 82 46%	Recommended: n/a Actual: 130 32.5%	PNV, 600 mg calcium, Vit D 1000 IU, choline, carnitine 300 mg, riboflavin 200 mg BID, Fe 15 mg (every other day)

**Table 2:** Intrapartum and postpartum MADD management checklist.

Intrapartum: ▪ Upon admission, obtain glucose, BMP, AST, ALT, CPK. ▪ Upon admission, notify metabolic genetics, cardiology, and obstetric anesthesia. ▪ Obtain glucose, CPK every 4 h or as clinically indicated. ▪ Obtain 12-lead ECG upon admission and start continuous cardiac telemetry. ▪ Continue enteral intake restricted in fat and protein, goal 65–75 g protein/day and 65–75 g fat/day (1 g/kg/d). ▪ Continue riboflavin 200 mg PO BID (unless NPO). ▪ Continue enteral intake as long as possible with frequent snacks to avoid prolonged fasting. ▪ Replete electrolytes as needed, either IV or PO. ▪ If patient develops altered mental status, obtain STAT ammonia, ABG, BMP, AST, ALT, CPK and page metabolic genetics. ▪ If chorioamnionitis or other infection develops, treat promptly with antibiotics and antipyretics, and start dextrose as below. ▪ Any of the following may precipitate catabolism; start dextrose containing IV fluids immediately for: emesis, fever, chorioamnionitis, active labor, epidural placement, decreased PO intake, or functional NPO status. ○ IV 10% dextrose (D10) in normal saline with 10 mEq KCl/L at 1.5xx maintenance; ○ IV Clinimix (4.25/5) or other IV amino acid mix at 1 g/kg total protein per day; ○ IV carnitine at 20–40 mg/kg/24 h (continuous or divided QID).
Postpartum: ▪ Resume diet restricted in fat and protein as above, resume metabolic supplements including oral carnitine and riboflavin. ▪ Plan ahead with hospital food services to provide snack and meal availability 24/7 while admitted. ▪ Continue IV dextrose and Clinimix until clearly tolerating enteral intake. ▪ If no arrhythmias present, may discontinue telemetry once tolerating enteral intake. ▪ Obtain glucose, BMP, AST, ALT, CPK 4 h postpartum, then postpartum day 1. May discontinue lab monitoring if labs above are normal. ▪ If breastfeeding or lactating, maintain caloric intake at 25% above pre-pregnancy levels (additional 330–500 kcal/day). ○ Protein: ~0.8 g/day + 25 g/d = 60–70 g/d (based on increased needs for lactation); ○ Fat: ~55–65 g/d (based on ~25–30% kcal from fat, ~1 g/kg/d). ▪ Monitor for changes in mood and appetite.
Legend: BMP= basic metabolic panel, ABG=arterial blood gas, AST=aspartate transferase, ALT= alanine transaminase, CPK= creatine phosphokinase, ECG= electrocardiogram.

## Data Availability

The data presented in this study are available on request from the corresponding author due to privacy restrictions.
